# Supplementary material for: Baseline Characterization of the Gut Microbiota of Field and Colony Populations of Phlebotomus tobbi and Preliminary Assessment of the Anti-Leishmanial Activity of Cultivable Bacteria
Source: Pathogens. 2026 Jun 23;15(7):658. doi: 10.3390/pathogens15070658 (PMC13416292; doi:10.3390/pathogens15070658)
Supplement: Supplementary file 1 [file pathogens-15-00658-s001.zip › pathogens-4357168-File S1/Composition_Barplots/Figures/charts/gBP57NeeBScZzFJ9Z1hsjz7tZHWt5K_legend.pdf]

k__Bacteria;p__Actinobacteria;c__Actinobacteria
k__Bacteria;p__Firmicutes;c__Bacilli
k__Bacteria;p__Firmicutes;c__Clostridia
k__Bacteria;p__Proteobacteria;c__Alphaproteobacteria
k__Bacteria;p__Proteobacteria;c__Betaproteobacteria
k__Bacteria;p__Proteobacteria;c__Gammaproteobacteria
